# Stable and quantitative small-scale laboratory propagation of *Cryptocaryon irritans*

**DOI:** 10.1016/j.mex.2020.101000

**Published:** 2020-07-19

**Authors:** Yuho Watanabe, Kah Hui How, Kosuke Zenke, Naoki Itoh, Tomoyoshi Yoshinaga

**Affiliations:** a Department of Aquatic Bioscience, Graduate School of Agriculture and Life Sciences, The University of Tokyo, 1-1-1, Yayoi, Bunkyo-ku, Tokyo, 113-8657, Japan

**Keywords:** *In vivo*, Controlled challenge, *Poecilia* sp, Infection

## Abstract

We established a laboratory propagation method of *Cryptocaryon irritans*, a parasitic ciliate of marine fishes, with black molly *Poecilia* sp. as host fish, using small plastic aquaria. One cycle of the propagation usually takes one week. With this method, 1500–3000 protomonts are obtained from five challenged mollies every week, from which more than 100,000–200,000 theronts are obtained. Using this method, an isolate of *C. irritans* has been successfully maintained more than three years. This propagation method reduces labor for maintaining and propagating the parasite and will much contribute to researches on cryptocaryoniasis.

• The method is a laboratory propagation technique of *Cryptocaryon irritans*.
• Using small plastic aquaria and black molly as a host, the parasites can be stably propagated and maintained.
• An isolate of *C. irritans* has been successfully maintained more than three years.

## Graphical abstract

**Figure:**
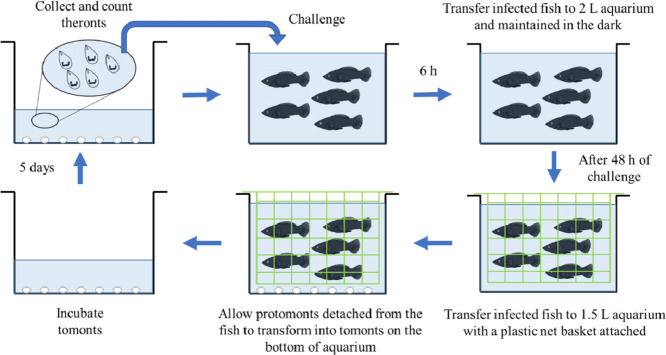

Specifications Table

**Table:** 

Subject Area	Agricultural and Biological Sciences
More specific subject area	Fish parasitology
Method name	*In vivo* propagation method of *Cryptocaryon irritans*
Name and reference of original method	Our method is a modified version of the method described in the article Yoshinaga T., Dickerson H.W. (1994). Laboratory propagation of *Cryptocaryon irritans* on a saltwater-adapted *Poecilia* hybrid, the black molly, Journal of Aquatic Animal Health 6(3) 197–201.Watanabe Y., How K.H., Zenke K., Itoh N., Yoshinaga T. (2020). Control of the daily rhythms by photoperiods in protomont detachment and theront excystment of the parasitic ciliate *Cryptocaryon irritans*, Fish Pathology 55 (2) 38–41.
Resource availability	*none*

## Background

*Cryptocaryon irritans* Brown 1951 is an obligate parasite of marine fishes with a quadriphasic lifecycle consisting of the infective theront stage, parasitic trophont stage, external protomont stage, and reproductive tomont stage [1], [2], [3]. This parasite was first reported in marine fishes of public aquaria and hobbyists [1,^4^,5] but later has been reported as one of major obstacles in warm-water marine fish culture [2,^3^,5]. Infection with *C. irritans* damages the hosts’ epidermis of the skin and gills of fish hosts, and disrupts their osmoregulation and respiration activity. Additionally, intensive culture in confined spaces eventually leads to heavy infection, often causing mass mortalities and posing major economic damage [6].

In order to mitigate the impact of this parasite on aquaria and mariculture, intensive and continuous studies using laboratory isolates propagated and maintained long are required; however, difficulties in long and stable propagation of the parasite prevent the progress of studies needed. Most of experimental studies on the parasite have been carried out using the parasite temporarily propagated on fish hosts [7,8], which required much seawater and relatively large fish rearing facilities. A small-scale propagation method was previously described [9], in which the parasite was passaged on seawater-adapted *Poecilia* sp. (black molly) by adding naïve fish in 50–150 L seawater propagation aquaria with a biological filter at intervals and harvesting infected fish from the aquaria with some infected fish left for next-round infection. This method has advantages that commercially supplied freshwater black molly without history of previous infection with the parasite are used after acclimatization to seawater and that relatively small size of aquaria are required. We have been using this method for more than 10 years for the propagation; however, this method is also neither stable nor quantitative, and excessive or low infection often leads to loss of infected fish and the parasite from propagation aquaria. To our best knowledge, continuous and stable propagation of the parasite for long periods has not been achieved yet. An*in vitro* culture method of the parasite was previously developed [10], in which trophonts can be grown to protomonts using cultured fish cells as feed; however, it is still impossible to propagate and keep the parasite continuously due to low recovery percentages of protomonts. Here, we developed a small-scale, quantitative and stable propagation method to passage *C. irritans* on seawater-adapted black molly using small plastic aquaria (2 L), which enables long-period propagation of the parasite with high yield of theronts needed for experiments in laboratories.

## Materials and equipments

• Naïve seawater-adapted *Poecilia* sp. (black molly)(3–4 cm body length; 0.7–1.5 g body weight)
• Filtered seawater (5.0 *μ*m, 30–35‰)
• Filter-sterilized seawater (0.22 *μ*m, 30–35‰)
• Antibiotics mixture (50,000 IU/mL penicillin G potassium and 50,000 µg /mL streptomycin sulfate)
• Plastic aquaria (2.0 L and 1.0 L)
• Plastic net basket (mesh opening 6.4 × 6.4 mm)
• Incubator with a chamber big enough to accommodate 2 L plastic aquaria
• 96-well plates for theront counting
• Inverted microscope for theront counting

## Procedure

1. Obtain theronts of *C. irritans* propagated on seawater-adapted black molly in a seawater aquarium equipped with a biological filter according to Yoshinaga et al., 1994 [9].

Notes: If *C. irritans* has not been propagated yet, obtain infected ornamental or food fish from a local pet shop or a fish farm as a source of infection. Place them in a filtered-seawater aquarium of adequate size overnight to obtain protomonts detached from the fish. Wash the obtained protomonts with filter-sterilized seawater supplemented with the antibiotics mixture (final concentrations: 500 IU/mL penicillin G potassium and 500 *μ*g/mL streptomycin sulfate) and incubate them in the filer-sterilized seawater with antibiotics at 25̊ C to obtain theronts according to Yoshinaga et al., 1994 [9].

2. Place five naïve black mollies (3–4 cm, 0.7–1.5 g) with 5000 theronts (1000 theronts/fish) in 1.5 L of filtered seawater in a 2 L plastic aquarium at 25 °C in the dark in an incubator for 6 h for challenge. Whole the process of fish rearing and incubation of tomonts should be carried out at 25̊ C.

Notes: Use naïve fish always for challenge, for fish once infected with *C. irritans* can acquire protective immunity against its infection as previously reported [4,^11^,12].

3. Transfer the challenged black mollies in 1.5 L of fresh filtered seawater in another 2 L plastic aquarium and keep them there in the dark with gentle aeration.
4. Forty-eight hours after the end of the challenge, when trophonts of *C. irritans* become visible by naked eyes as pinhead white spots on the surface of the skin and fins of fish, transfer the fish into a plastic net basket set in 1.0 L filtered seawater in a 1.5 L plastic aquarium in the dark in the incubator.
5. During the next 24 h, allow the protomonts to be detached from fish, settle and transform into encysted tomonts attaching to the bottom of the aquaria. Subsequently, remove the fish and basket from the aquaria.
6. Rinse the bottom of aquarium with filter-sterilized seawater supplemented with antibiotics mixture (final concentrations: 500 IU/mL penicillin G potassium and 500 μg/mL streptomycin sulfate) three times and place the aquarium in an incubator, with 50 mL filter-sterilized seawater left. Give 12 h light and 12 h dark photoperiod in the incubator (6:00–18:00 Light, 18:00–6:00 Dark). Replace the seawater in the aquarium with fresh one every day.
7. Five to seven days after the step 6, when tomonts release theronts mostly from 6 to 3 h prior to the end of the dark period (see additional information), collect theront suspension in the aquarium. Determine the concentration of theronts by counting them in 50 *µ*L aliquot of the suspension five times in 96-well plates under an inverted microscope and use the theronts for next round of infection or experiments.
8. For next round of infection, go to the step 2. Repeat the steps 2 to 7 for routine propagation of *C. irritans.*

## Method validation

**Fig. 1:**
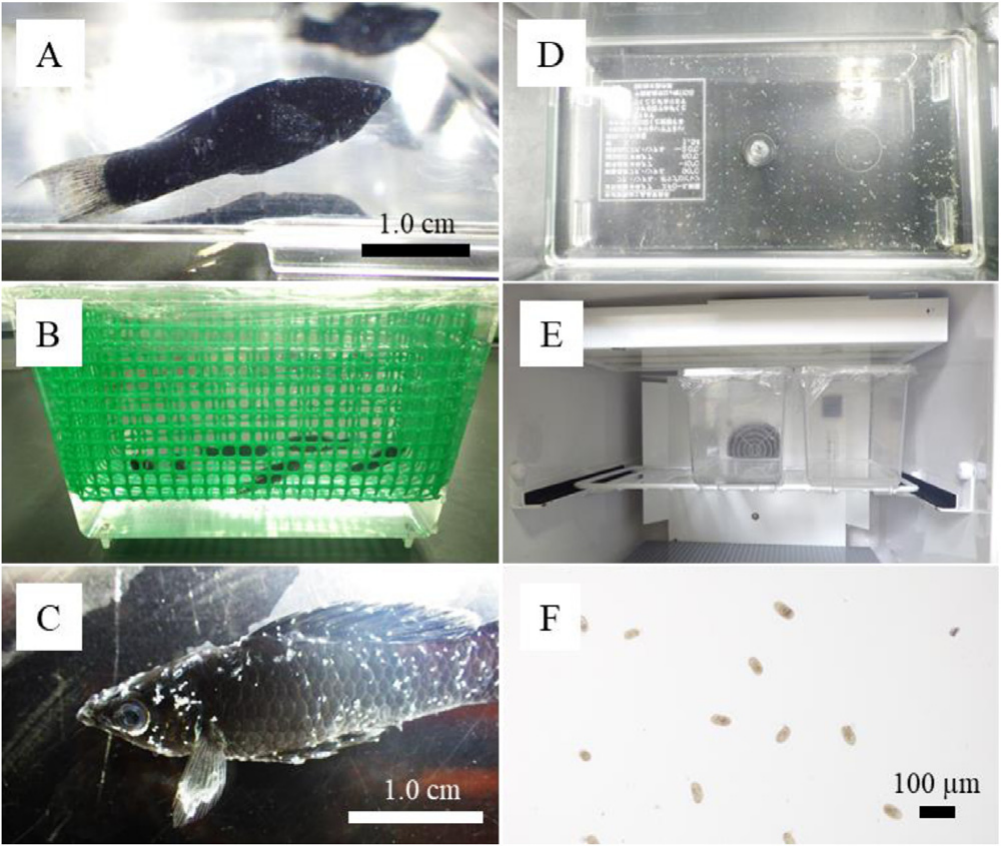
Procedure of the propagation method of *Cryptocaryon irritans.* A: Trophonts becoming visible on fish 48 h after the end of challenge. B: Infected fish accommodated in a plastic net cage in a 1.5 L plastic aquarium. C: Trophonts fully developed to be detached from infected fish. D: Clusters of tomonts attached to the bottom of an aquarium. E: Tomont incubation in an incubator F: Theronts released from tomonts.

We use the method to maintain and propagate the parasite routinely. Each round of infection usually takes 7 days post challenge. Trophonts become visible on fish usually 48 h after the end of challenge (Fig. 1A). During the next 24 h, most trophonts fully develop to be detached from fish as protomonts (Fig. 1B). A total of 1500–3000 protomonts are recovered from five infected fish (300–600 protomonts/fish) challenged with 5000 theronts in total; the recovery is estimated to be 30–60%. Theronts begin to excyst from tomonts 4 days after detachment from the fish (Fig. 1C). Theront excystment peaks 5 days after detachment. Total number of theronts obtained from each aquarium is approximately 100,000–200,000. At this time point, the fresh theronts within 3 h after excystment are collected for the next cycle. By this method, we have succeeded in propagating and maintaining an isolate of *C. irritans* for more than three years. As the equipment and space needed for propagation are not much, the method will make it possible to hold plural isolates in a laboratory, which enable us comparison of biology among different isolates of the parasite, although much attention is still necessary to avoid cross contamination.

## Conclusion

By repeating controlled challenges with *Cryptocaryon irritans* on small number of black mollies seawater-adapted *Poecilia* sp. using small plastic aquaria, stable and quantitative propagation can be achieved continuously.

## CRediT authorship contribution statement

**Yuho Watanabe:** Conceptualization, Investigation, Writing - original draft. **Kah Hui How:** Conceptualization. **Kosuke Zenke:** Conceptualization, Investigation. **Naoki Itoh:** Supervision. **Tomoyoshi Yoshinaga:** Supervision, Funding acquisition, Writing - review & editing.

## Declaration of Competing Interest

The authors declare that they have no known competing financial interests or personal relationships that could have appeared to influence the work reported in this paper.

## References

[1] Sikama Y. (1997). Preliminary report on the white-spot disease in marine fishes. Suisan-gakukai-ho.

[2] Colorni A. (1985). Aspects of the biology of *Cryptocaryon irritans*, and hyposalinity as a control measure in cultured gilt head sea bream Sparus aurata. Dis. Aquat. Org..

[3] Colorni A., Burgess P. (1997). *Cryptocaryon irritans* Brown 1951, the cause of ‘white spot disease’ in marine fish: an update. Aquar. Sci. Conserv..

[4] Dickerson H.W. (2006). *Ichthyophthirius multifiliis* and *Cryptocaryon irritans* (Phylum Ciliophora), Fish Diseases and Disorders.

[5] Wilkie D.W., Gordin H. (1969). Outbreak of cryptocaryoniasis in marine aquaria at Scripps Institution of Oceanography. Calif. Fish Game.

[6] Colorni A. (1987). Biology of *Cryptocaryon irritans* and strategies for its control. Aquaculture.

[7] Dan X.M., Li A.X., Lin X.T., Teng N., Zhu X.Q. (2006). A standardized method to propagate *Cryptocaryon irritans* on a susceptible host pompano *Trachinotus ovatus*. Aquaculture.

[8] Burgess P.J., Matthews R.A. (1994). A standardized method for the in-vivo maintenance of *Cryptocaryon irritans* (Ciliophora) using the gray mullet *Chelon labrosus* as an experimental host. J. Parasitol..

[9] Yoshinaga T., Dickerson H.W. (1994). Laboratory propagation of *Cryptocaryon irritans* on a saltwater-adapted *Poecilia* Hybrid, the black molly. J. Aquat. Anim. Health.

[10] Yoshinaga T., Akiyama K., Nishida S., Nakane M., Ogawa K., Hirose H. (2007). In vitro culture technique for *Cryptocaryon irritans*, a parasitic ciliate of marine teleosts. Dis. Aquat. Org..

[11] Burgess P.J., Matthews R.A. (1995). *Cryptocaryon irritans* (Ciliophora): acquired protective immunity in the thick-lipped mullet, *Chelon labrosus*. Fish Shellfish Immunol..

[12] Yoshinaga T., Nakazoe J.I. (1997). Acquired protection and production of immobilization antibody against *Cryptocaryon irritans* (Ciliophora, Hymenostomatida) in Mummichog (*Fundulus heteroclitus*). Fish Pathol..

[13] Watanabe Y., How K.H., Zenke K., Itoh N., Yoshinaga T. (2020). Control of the daily rhythms by photoperiods in protomont detachment and theront excystment of the parasitic ciliate *Cryptocaryon irritans*. Fish Pathol..

